# Study on Circulating Antigens in Serum of Mice With Experimental Acute Toxoplasmosis

**DOI:** 10.3389/fmicb.2020.612252

**Published:** 2021-01-18

**Authors:** Qi Liu, Wei Jiang, Yun Chen, Manyu Zhang, Xiaoling Geng, Quan Wang

**Affiliations:** Shanghai Veterinary Research Institute, Chinese Academy of Agricultural Sciences, Shanghai, China

**Keywords:** *Toxoplasma gondii*, acute infection, circulating antigens, proteomics, diagnostic candidates

## Abstract

*Toxoplasma gondii* is a ubiquitous apicomplexan protozoan parasite that can infect all warm-blooded animals, causing toxoplasmosis. Thus, efficient diagnosis methods for acute *T. gondii* infection are essential for its management. Circulating antigens (CAgs) are reliable diagnostic indicators of acute infection. In this study, we established a mouse model of acute *T. gondii* infection and explored new potential diagnostic factors. CAgs levels peaked 60 h after *T. gondii* inoculation and 31 CAgs were identified by immunoprecipitation-liquid chromatography-tandem mass spectrometry, among which RuvB-like helicase (TgRuvBL1), ribonuclease (TgRNaseH1), and ribosomal protein RPS2 (TgRPS2) were selected for prokaryotic expression. Polyclonal antibodies against these three proteins were prepared. Results from indirect enzyme-linked immunosorbent assay indicated that anti-rTgRuvBL1, anti-rTgRNase H1, and anti-rTgRPS2 mouse sera were recognized by natural excretory-secretory antigens from *T. gondii* tachyzoites. Moreover, immunofluorescence assays revealed that TgRuvBL1 was localized in the nucleus, while TgRNase H1 and TgRPS2 were in the apical end. Western blotting data confirmed the presence of the three proteins in the sera of the infected mice. Moreover, mice immunized with rTgRuvBL1 (10.0 ± 0.30 days), TgRNaseH1 (9.67 ± 0.14 days), or rTgRPS2 (11.5 ± 0.34 days) had slightly longer lifespan when challenged with a virulent *T. gondii* RH strain. Altogether, these findings indicate that these three proteins can potentially be diagnostic candidates for acute toxoplasmosis. However, they hold poor protective potential against highly virulent *T. gondii* infection.

## Introduction

*Toxoplasma gondii* is an obligatory intracellular protozoan parasite that infects virtually any warm-blooded animal, including a third of the human population (Montoya and Liesenfeld, 2004). Infection is mainly acquired by ingestion of undercooked meats containing *T. gondii* cysts (Hide et al., 2009). After ingestion, the parasite causes lifelong infections by converting from rapidly dividing tachyzoites into encysted slow-growing bradyzoites, mainly localizing to the brain, and muscle tissues (Hunter and Sibley, 2012; Wohlfert et al., 2017). Moreover, a latent infection may be reactivated via immune suppression (Dupont et al., 2020). In general, toxoplasmosis is usually asymptomatic in immunocompetent persons, but it can cause life-threatening infections in immunocompromised individuals and developing fetuses (Nissapatorn, 2009; Csep and Drãghici, 2013; Lima and Lodoen, 2019). Thus, timely and effective diagnosis of acute *T. gondii* infection is imperative.

Convenient serological tests have been widely applied to diagnose toxoplasmosis, which are mainly based on the detection of specific antibodies (IgM and IgG; Robert-Gangneux and Dardé, 2012). IgG can only be detected 13 days after infection, whereas IgM can persist in some patients with toxoplasmosis for over a year (Elsheikha et al., 2020). Thus, the presence of IgG and IgM antibodies does not necessarily indicate an acute infection. The lack of effective serological diagnostic methods for acute toxoplasmosis can lead to delayed treatment and even therapy failure, since available strategies can control acute infections, but not treat chronic toxoplasmosis.

*Toxoplasma gondii* excretory-secretory antigens (ESA) represent the majority of circulating antigens (CAgs) in the sera of hosts with acute toxoplasmosis (Daryani et al., 2003; Abdollahi et al., 2009). Previous studies have shown that *T. gondii* CAgs can be used as accurate markers for acute toxoplasmosis diagnosis (Hafid et al., 1995; Meira et al., 2008; Abdollahi et al., 2009; Darcy et al., 2010; Xue et al., 2016). Therefore, analysis of CAg profiles and screening of candidate diagnostic molecules should improve the specificity and sensitivity of detection methods for acute infection. Moreover, CAgs modulate the host immune response (Hafid et al., 2005; Abdollahi et al., 2013; Daryani et al., 2014; Carlos et al., 2019; Xue et al., 2019) and the identification of CAg components may be conducive to the discovery of potential candidate antigens for vaccines against toxoplasmosis. To date, CAgs have not been investigated in detail, and only a few CAgs components have been identified. The present study aimed to determine the spectrum of *T. gondii* CAgs in sera of acute infected mice using immunoprecipitation-liquid chromatography (LC)-tandem mass spectrometry (MS/MS). We also aimed to verify three novel CAgs in the sera of infected model mice and to evaluate their protective ability.

## Materials and Methods

### Parasite Strains and Culture

The *T. gondii* strain RH and African green monkey kidney (VERO) cells were stored at our facility. *T. gondii* tachyzoites were grown in VERO cells maintained in Dulbecco’s Modified Eagle’s Medium (DMEM; Gibco Laboratories, Gaithersburg, MD, United States) supplemented with 2% fetal bovine serum, 100 kU/L streptomycin, and 400 kU/L penicillin at 37°C under a 5% CO_2_ atmosphere.

### Animals

Eight-week old Kunming mice and New Zealand white female rabbits weighing ∼25 *g* and ∼2 kg, respectively (Shanghai Laboratory Animal Centre, Chinese Academy of Sciences, Shanghai, China) were raised in a sterilized room and fed with sterilized food and water at the Animal Laboratory Centre at the Shanghai Veterinary Research Institute. The Animal Care and Use Committee of the Shanghai Veterinary Research Institute approved the study and the protocols complied with approved guidelines.

### ESA and Antisera Preparation

Excretory-secretory antigens were obtained as previously described (Zhou et al., 2005). Briefly, purified tachyzoites (5 × 10^8^/mL) suspended in phosphate-buffered saline (PBS) were incubated at 37°C for 2 h. After centrifugation for 15 min at 1,000 × *g* at 4°C, the supernatant was collected, and the protein density was determined using DC protein assay kits (Bio-Rad Laboratories Inc., Hercules, CA, United States). Thereafter, 1 mg of protein per tube (1.5 mL) was lyophilized. At 0, 2, 4, and 6 weeks, the mice and rabbits were immunized with 100 μg per mouse and 500 μg/kg proteins, respectively, or PBS (the control). The 206 adjuvant was applied as described by the manufacturer (Seppic, Paris, France). Blood was sampled from the tails of mice at the end of each interval for immunoblotting and enzyme-linked immunosorbent assay (ELISA).

### Establishment of Double-PcAb Sandwich ELISA

A double-PcAb sandwich ELISA was established to detect CAgs using anti-ESA rabbit and mouse sera as coating and detection antibodies, respectively. Nonspecific protein binding was blocked with PBS containing 0.05% Tween-20 (PBST) and 5% non-fat powdered milk. Briefly, Costar microtiter plates (Corning Inc., Corning, NY, United States) were coated with anti-ESA rabbit serum in 50 mM carbonate buffer overnight at 4°C, then washed with PBST. Nonspecific binding was blocked for 2 h at 37°C. The plates were washed, then 500 ng/mL ESA or samples were added and the plates were incubated for 1 h at 37°C, and washed. Sample dilution buffer was the negative control. Anti-ESA mouse sera were added to the plates and incubated for 1 h at 37°C, followed by three washes with PBST. Secondary HRP-conjugated goat anti-mouse IgG antibody (Cat. No. SE131; Solarbio, Beijing, China) was added, and the plates were incubated for 1 h at 37°C. After five washes with PBST, immune complexes were visualized by incubation with tetramethylbenzidine (TMB) for 20 min. The reaction was stopped by adding 2 M H_2_SO_4_, and absorbance was measured at 450 nm using an automated MULTISKAN GO microplate reader (Thermo Fisher Scientific Inc., Waltham, MA, United States). All samples were analyzed in triplicate. The optimal coating and detection concentrations were determined using checkerboard titration. The sensitivity of the method was assessed using positive canine sera at 3 days post-challenge with *T. gondii* (Xue et al., 2016).

### Development of Acute Infection Models

Control and experimental (*n* = 3 each) mice were injected with 500 μL of PBS without and with 1 × 10^7^ purified tachyzoites, respectively. Blood (100 μL) samples were collected at 0, 3, 9, 12, 24, 36, 48, and 60 h. Portions of these samples (20 μL) were mixed with sodium citrate anticoagulant for genomic DNA extraction and *T. gondii* detection, and serum CAgs was detected and purified by immunoprecipitation in 80 μL of samples.

### Evaluation of Infection Models Using ELISA and Nested PCR

We analyzed CAgs in mouse sera using the double-PcAb sandwich ELISA. To detect *T. gondii*, DNA was obtained from anticoagulated blood using a genomic DNA extraction kit (Cat. No. DP308; Tiangen, Beijing, China). Nested PCR was conducted as we previously described (Wang et al., 2012).

### Immunoprecipitation

Proteins of interest were immunoprecipitated as described (Xue et al., 2016), mixed with sera at 60 h after challenge (20 μL per mouse), and diluted (1:10) in PBS. Protein G agarose beads (200 μL, 25%; Cat. No. P2009; Beyotime, Nantong, China) were added, and stirred at 4°C for 3 h to remove serum antibodies. The supernatant was collected after centrifugation (260 × *g*, 4°C, 5 min), then 10 μL of anti-ESA mouse serum was added, and stirred slowly overnight at 4°C. Protein G agarose beads (200 μL) were added, and the mixture was stirred at 4°C for 3 h. The beads were collected by centrifugation (260 × *g*, 4°C, 5 min), washed three times with PBS, and boiled in 5× sample buffer (60 μL) for 5 min. The supernatant collected after centrifugation (260 × *g*, 4°C, 5 min) was resolved by 10% sodium dodecyl sulfate-polyacrylamide gel electrophoresis (SDS-PAGE), then analyzed by LC-tandem mass spectrometry (LC-MS/MS; Shanghai Applied Protein Technology Co., Ltd., Shanghai, China).

### LC-Electrospray Lonization MS/MS Analysis

Samples were analyzed using a Q Exactive mass spectrometer coupled with an Easy-nLC (Thermo Fisher Scientific Inc.) liquid chromatograph. Briefly, samples were reduced, alkylated, and trypsinized (mass ratio 1:50) at 37°C for 20 h. The enzymolysis product was desalted, lyophilized, dissolved in 0.1% formic acid, and stored at -20°C. For MS analysis, solution A comprised 0.1% formic acid in high performance liquid chromatography (HPLC)-grade water, and solution B comprised 0.1% formic acid in 84% acetonitrile. After the chromatographic column was equilibrated with 95% solution A, samples were loaded from the autosampler to the trap column (0.5 H gradient). To collect mass spectra, the mass-to-charge ratios of peptides and peptide fragments were determined by collecting 20 fragment maps (MS2 scan) after each full scan. The original raw files from the MS test were used to search the UniProt database for proteins using Mascot 2.2 software. The search parameters were as follows: enzyme, trypsin; fixed modification, carbamidomethyl (C); variable modification, oxidation (M); missed cleavage, 2; peptide mass tolerance, 20 ppm; MS/MS tolerance, 0.1 Da; and filter by score ≥ 20.

### Expression of TgRuvBL1, TgRNase H1, and TgRPS2 *in vitro* and Antibody Production

To further verify the CAgs components in serum, RuvB-like helicase (TgRuvBL1), ribonuclease (TgRNase H1), and ribosomal protein RPS2 (TgRPS2) were expressed in an *Escherichia coli* (BL 21) system with the pET-28a-c(+) vector. Table 1 shows the expressed regions, and primers, etc. The sequence of the recombinant plasmid was confirmed by dideoxy chain termination sequencing (Invitrogen, Carlsbad, CA, United States). The recombinant plasmid was transformed into *E. coli* (BL21) competent cells, and cultured in LB medium containing kanamycin (70 μg/mL) at 37°C. Expression was induced with 1 mM isopropyl β-D-thiogalactopyranoside (IPTG) for 6 h at 37°C. Cells were sonicated, then recombinant proteins isolated from inclusion bodies were refolded using TB234 kits (Cat. No: 70123-3; Novagen, San Diego, CA, United States), and purified using His-tag protein purification kits (Cat. No. P2226; Beyotime, Nantong, China). Recombinant proteins were concentrated using 3-kDa centrifugal filters (Merck KGaA, Darmstadt, Germany), and quantified using DC protein assay kits (Bio-Rad Laboratories Inc.). At 0, 2, and 4 weeks, the mice were injected with each protein (100 μg per mouse) or PBS (control). Blood samples were collected from the tail vein of mice at the end of each interval for indirect ELISA and immunofluorescence assay analysis. For indirect ELISA, plates were coated with recombinant proteins (500 ng/mL) or ESA (1 μg/mL). Horseradish peroxidase-conjugated goat anti-mouse IgG were diluted 1:5,000.

**TABLE 1:** Information of expressed protein.

Protein name	Expressed region	Primers^a^	Restriction enzyme	Form of expression	Recombinant protein weight (Da)	Antiserum dilution
TgRuvBL1	Overall length	F:5′-ACCGGATCCATGGAGCAGSCCGCTCGTGGAG-3′	Bamh I	Inclusion body	41.98 kDa	1:32000
		R: 5′-ACCCTCGAGCTCATCCATGAAAAGGACCCC-3′	Xho I			
TgRNase H1	Overall length	F:5′-ACCGGATCC CGGGAGAGAAGCGAGGCGCCA-3′	Bamh I	Inclusion body	37.52 kDa	1:32000
		R: 5′-ACC GCGGCCGC TTAAACGCCCAGSGTTGGAGAG-3′	Not I			
TgRPS2	Overall length	F:5′-ACCGGATCCATGGCAGSAACGCGGCAGSCTTT-3′>	Bamh I	Inclusion body	35.34 kDa	1:32000
		R: 5′-ACCCTCGAGCTAGGCGAGCGGTCGCGACAC-3′	Xho I			

*^*a*^Enzyme cleavage sites were underlined.*

### Immunofluorescence Assays

For Immunofluorescence assays (IFA), infected confluent VERO monolayers were fixed with 4% paraformaldehyde in PBS, permeabilized with 0.5% Triton X-100, and blocked with 1% bovine serum albumin in PBS (BSA-PBS). The primary antibodies used were mouse anti-TgRuvBL1 (1:500), mouse anti-TgRNase H1 (1:500), mouse anti-TgRPS2 (1:500), and rabbit anti-SAG1 (1:4,000). The secondary antibodies used were Alexa Fluor 488-conjugated goat anti-mouse IgG (1:2,000; Thermo Fisher Scientific Inc.) and Alexa Fluor 594-conjugated goat anti-rabbit IgG (1:2,000; Jackson ImmunoResearch, West Grove, PA, United States). Images were acquired with a LSM880 confocal laser scanning microscope (Zeiss, Germany) after the samples were stained with DAPI.

### Verification of TgRuvBL1, TgRNase H1, and TgRPS2 in Sera of Acute Infection Model

To verify the presence of TgRuvBL1, TgRNase H1, and TgRPS2 in the sera of infected model. Protein G agarose beads were used to eliminate serum antibodies, then the pooled sera (60 h) from the test and control (negative) group were analyzed by immunoblotting. The positive control was ESA. Primary TgRuvBL1, TgRNase H1, and TgRPS2 antisera were diluted 1:1000, and HRP-conjugated goat anti-mouse IgG secondary antibody was diluted 1:5 000.

### Mouse Vaccination and Challenge

To further explore the protective ability of TgRuvBL1, TgRNase H1, and TgRPS2, 48 mice were randomly divided into four groups and were subcutaneously injected at weeks 0, 2, and 4 with 100 μg of rTgRuvBL1, rTgRNase H1, rTgRPS2, or PBS (control group), all with 206 adjuvant. Blood was sampled from all mice on week 6. Anti-ESA IgG was determined by ELISA in mice from each group. Afterward, 12 mice from each group were intraperitoneally challenged with 1 × 10^3^ tachyzoites of the *T. gondii* RH strain.

### Statistical Analysis

All data were statistically analyzed using IBM SPSS 20.0 Data Editor. Differences in data among all groups were compared using one-way ANOVA. Survival of the mice was compared using Kaplan–Meier curves. Differences between groups were considered significant at *P* < 0.05.

## Results

### Establishment of Double-PcAb Sandwich ELISA

**FIGURE 1:**
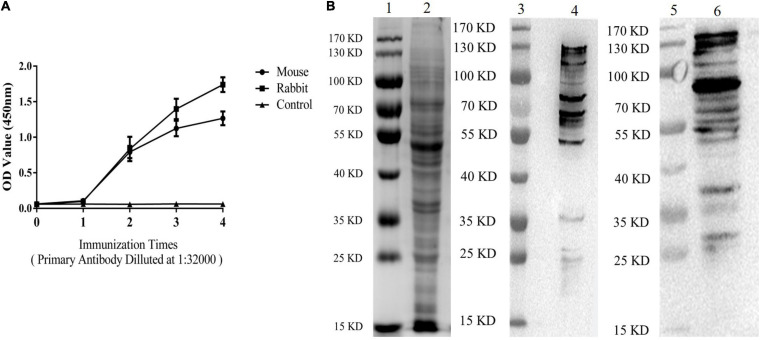
ESA antibodies were tested by ELISA and immunoblotting analyses. **(A)** Levels of ESA antibodies after each immunization (Mouse and Rabbit). Blood sera were sampled from the tails of mice and the ear vein of rabbit 10 days after immunization. **(B)** Western blotting analysis of ESA. Lane 1: marker on gel; Lane 2: ESA on gel; Lane 3: marker on NC membrane; and Lane 4: ESA on NC membrane (anti-ESA mice serum). Lane 5: marker on NC membrane; Lane 6: ESA on NC membrane (anti-ESA rabbit serum).

**FIGURE 2:**
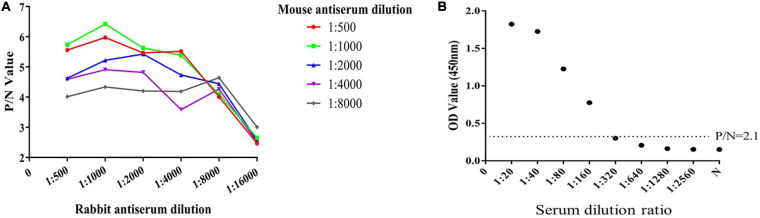
Determination of the optimum working concentration of mouse and rabbit antiserum and the maximum dilution of positive serum. **(A)** The optimal working coating concentration (anti-ESA rabbit serum) and detection concentration (anti-ESA mice serum) were determined using the checkerboard titration method. The P/N value was the criterion standard. **(B)** Sensitivity test using double-PcAb sandwich ELISA. The average optical density (OD) value for negative samples was multiplied by 2.1 to obtain the cut-off value. The horizontal lines represent cut-off values.

The results of indirect ELISA and immunoblotting assays indicated that anti-ESA mouse and rabbit sera could identify numerous ESA components with good immunogenicity (Figures 1A,B). According to the checkerboard titration results (Figure 2A), the P/N value was highest (6.42) at dilutions of 1:1 000 for both anti-ESA rabbit serum coating and mouse serum detection antibodies. The dilution gradient of positive canine sera was detected using the double antibody sandwich ELISA with a P/N cutoff of 2.1. Figure 2B shows that the maximum dilution of positive serum was 1:160. These results showed that the double-PcAb sandwich ELISA was sufficiently sensitive.

### Establishment of Murine *T. gondii* Infection Model Using Inoculation With Tachyzoites

**FIGURE 3:**
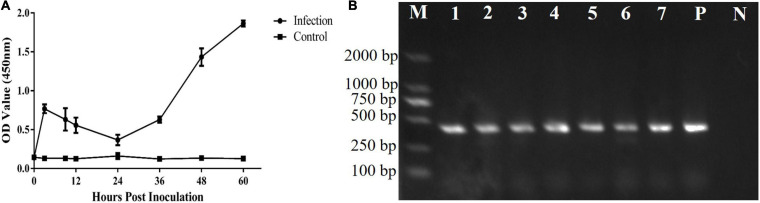
Analyses of CAgs and nested-PCR. **(A)** Sera of 0, 3, 9, 12, 24, 36, 48, and 60 h were tested for CAgs levels. **(B)** Nested-PCR results. Lane M: marker; Lane P: positive control; Lane N: negative control; and Lanes 1–7: Nested-PCR results of 3, 9, 12, 24, 36, 48, and 60 h, The nested-PCR results from one mouse in the test group are shown.

The *T. gondii* strain RH is lethal in mice. In this study, the mice survived for 65 ± 2 h after inoculation with 1 × 10^7^ tachyzoites. Both *T. gondii* and CAgs were detected in the blood at 3 h (Figures 3A,B). The CAgs values initially first increased from 0 to 3 h, decreased from 3 to 24 h, increased from (24 to 60 h), then peaked at 60 h. The decrease in CAgs from 3 to 24 h might have been due to *T. gondii* in the bloodstream invading various organs. These data showed that the *T. gondii* infection model was effective.

### Identification of Circulating Antigens in the Sera of Acute *T. gondii* Infected Mice

**FIGURE 4:**
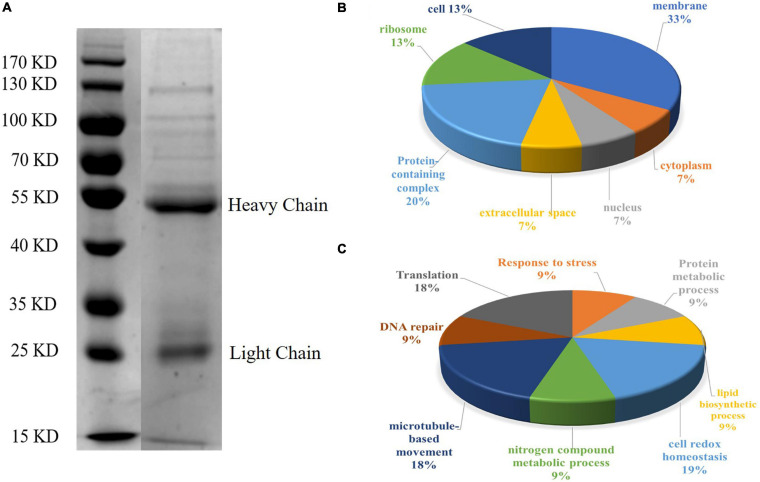
SDS-PAGE analysis and GO annotation of CAgs enriched and purified by immunoprecipitation. **(A)** Circulating antigens enriched and purified by immunoprecipitation were analyzed by SDS-PAGE (10%). Lane 1: marker; Lane 2: immunoprecipitation supernatant (In the original image, there is a crack in a lane near the marker, so it is cut off in the manuscript. Please refer to the original images in the Supplementary Figure 1). **(B)** Pie charts showing the GO distributions for the identified circulating antigens based on the cell component. **(C)** Pie charts showing the GO distributions for the identified circulating antigens based on the major biological process categories.

Sodium dodecyl sulfate-polyacrylamide gel electrophoresis analysis showed that CAgs weighted more than 15 kD (Figure 4A). Among 250 protein groups identified by LC-MS/MS (Supplementary File 1), the unique peptide counts for 31 proteins were ≥2. Table 2 shows that the proteins identified by immunoprecipitation-shotgun analysis comprised micronemal proteins (PLP1 and M2AP), surface antigens (SAG1), rhoptry protein (ROP28), dense granule proteins (PI-1), and novel CAgs proteins (RuvB-like helicase, ribonuclease, and ribosomal protein RPS2) and others (Table 2).

**TABLE 2:** CAgs proteins identified by LC-MS/MS after IP enrichment and purification with ESA mouse antibodies.

Resource ID	Protein ID	Protein name	Peptide count	Unique peptide count	Cover percent	Theoretical Mr (Da)/PI	References of analysis in *T. gondii*	GO_Term
1	S7UUF6	PLP1	21	18	17.74%	125493.06/6.01	Kafsack et al., 2009	Apical part of cell
2	A0A086JV38	MIC2-associated protein M2AP	2	2	2.12%	34551.68/4.28	Huynh et al., 2003	Membrane
3	B6D286	SAG1	2	2	12.72%	17922.19/6.46	Grimwood and Smith, 1992	Membrane
4	A0A2G8YCT9	Rhoptry protein ROP28	2	2	0.72%	78578.42/9.5	Jones et al., 2017	Binding
5	A0A2T6ITL9	Protease inhibitor PI1	2	2	1.37%	47808.7/5.06	Morris et al., 2002	Extracellular space
6	S7VNC9	Actin	8	7	23.00%	32058.23/5.66	Dobrowolski and Sibley, 1996	Binding
7	A0A086PH15	Histone H2A	2	2	10.32%	15919.54/10.63	Bogado et al., 2014	Binding
8	Q9NG25	Toxofilin	2	2	6.94%	27131.46/9.57	Poupel et al., 2000	Binding
9	A0A139XN72	Putative myosin regulatory light chain	2	2	5.83%	23123.97/6.86	Herm-Götz et al., 2002	Binding
10	B6KN45	Elongation factor 1-alpha	2	2	4.46%	49005.24/9.02	Wang et al., 2017	Binding
11	A0A086L4P6	Heat shock protein HSP70	2	2	4.35%	72291.98/5.07	Aosai et al., 2002	Response to stress
12	A0A086JS03	Putative transmembrane protein	2	2	2.13%	24896.27/6.85	Xue et al., 2016	Membrane
13	A0A2G8XVZ0	Carbamoylphosphate synthetase	2	2	1.82%	92154.94/8	Fox et al., 2009	Nitrogen compound metabolic process
14	A0A3R7YIU5	Phosphatidylinositol 3-and 4-kinase	2	2	1.44%	53688.12/8.86	Wengelnik et al., 2018	Kinase activity
15	A0A086LU85	Myosin H	2	2	1.42%	78984.17/8.82	Graindorge et al., 2016	Binding
16	A0A3R7YS28	CDPK9	2	2	1.31%	60638.79/7	Long et al., 2016	Binding
17	Q9BLM8	Protein disulfide-isomerase	2	2	1.27%	52801.38/5.14	Wang et al., 2013	Cell redox homeostasis
18	A0A086QXN6	Amine-terminal region of chorein	3	3	0.22%	1101135.3/8.74	Xue et al., 2016	Membrane
19	A0A086PFP4	HECT-domain (Ubiquitin-transferase) domain-containing protein	2	2	0.21%	998277.75/8.37	Xue et al., 2016	Protein metabolic process
20	A0A086KQL2	Putative type I fatty acid synthase	2	2	0.10%	530295.61/5.92	Zhu et al., 2000	Lipid biosynthetic process -
**Novel CAgs proteins**								
21	A0A086KTM5	RuvB-like helicase	2	2	8.57%	11770.33/5.64	Na	DNA repair
22	A0A086JWF3	Ribonuclease	2	2	4.16%	40446.13/9.59	Na	RNA-DNA hybrid ribonuclease activity
23	A0A086JV11	Uncharacterized protein	2	2	0.27%	433546.48/6.8	Na	Unknown
24	A0A139Y154	Ribosomal protein RPS2	2	2	2.23%	29335.57/10.21	Na	Translation
25	A0A3R8AB74	Ribosomal protein RPL7	2	2	1.94%	30121.23/10.37	Na	Translation
26	A0A425I0Y7	SAG-related sequence SRS55M	2	2	1.77%	41728.31/4.83	Na	Membrane
27	A0A2T6IQA3	Cation channel family transporter	2	2	1.04%	127269.78/8.94	Na	Binding
28	A0A086L241	Putative Chromosome-associated kinesin KLP1	2	2	0.79%	113375.72/4.99	Na	Microtubule-based movement
29	A0A151HDW0	Putative oxidoreductase	2	2	0.46%	146590.81/6.34	Na	Cell redox homeostasis
30	V4ZW70	SAG-related sequence SRS33	2	2	0.45%	607653.31/7.01	Na	Membrane
31	S8F1G6	Dynein heavy chain family protein	2	2	0.18%	446523/5.3	Na	Microtubule-based movement

### Gene Ontology Analysis

To further understand the functions of the CAgs herein identified, their gene ontology (GO) was evaluated. Figures 4B,C shows the GO annotations of the 31 high-confidence proteins, among which 15 were annotated in cell composition and were related to membrane components (33%), protein-containing complexes (20%), ribosomes (13%), cells (13%), extracellular regions (7%), the nucleus (7%), and the cytoplasm (7%). Only 11 proteins were annotated within biological process, being related to cell redox homeostasis (19%), translation (18%), microtubule-based movement (18%), response to stress (9%), protein metabolic processes (9%), lipid biosynthetic processes (9%), nitrogen compound metabolic processes (9%), and DNA repair (9%).

### Expression, Purification, and Antibody Production of Recombinant TgRuvBL1, TgRNase H1, and TgRPS2

**FIGURE 5:**
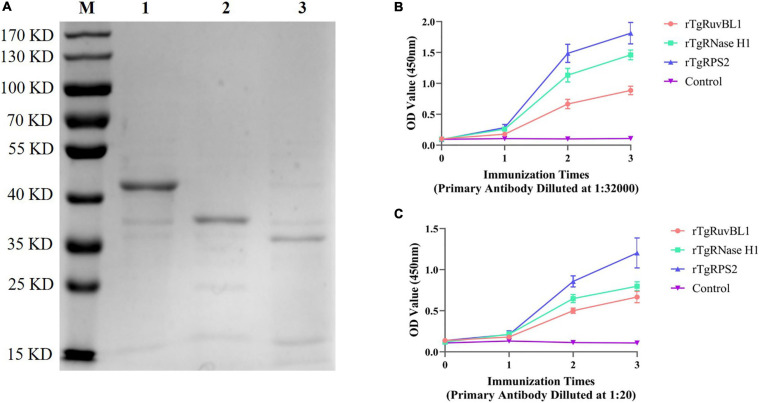
Expression of recombinant proteins and the dynamics of specific antibody amounts in Kunming mice induced by recombinant proteins. **(A)** SDS-PAGE analysis of purified recombinant proteins. M, Marker; 1, rTgRuvBL1; 2, rTgRNase H1; and 3, rTgRPS2. **(B,C)** Plates were coated with recombinant proteins (500 ng/mL) or ESA (1 μg/mL) to detect the dynamics of specific IgG antibody amounts.

The genes encoding TgRuvBL1, TgRNase H1, and TgRPS2 were amplified from the *T. gondii* RH strain. The recombinant proteins were expressed in *E. coli* as inclusion body His-tagged fusion proteins when bacterial growth occurred at 37°C. The inclusion body recombinant proteins were refolded, purified, and concentrated. SDS-PAGE analysis indicated that the recombinant proteins (rTgRuvBL1, rTgRNase H1, and rTgRPS2) were successfully expressed and purified (Figure 5A). Figure 5B shows that the titer of the antibodies in mice serum reached more than 1:32,000 after three immunizations.

### Reactivity of Polyclonal Antibodies Toward *T. gondii*-Derived ESA and Tachyzoites

**FIGURE 6:**
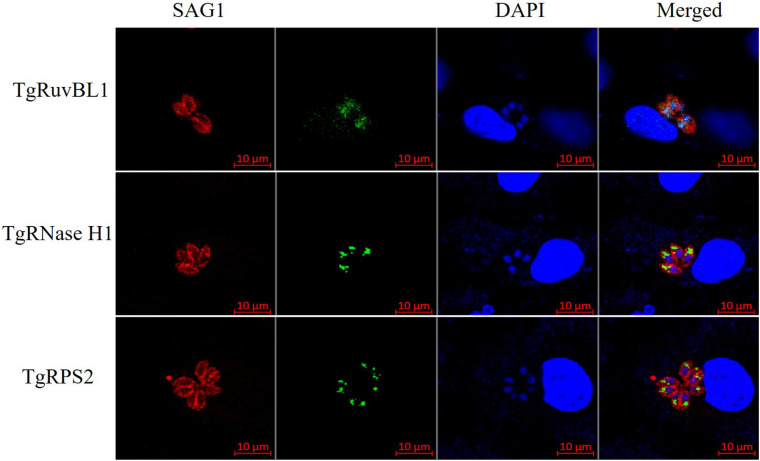
TgRuvBL1, TgRNase H1, and TgRPS2 localize to two subcellular regions. Immunofluorescence analysis showing the localization of the three CAgs, performed in *T. gondii* stained with anti-TgRuvBL1, anti-TgRNase H1, or anti-TgRPS2 antibodies. Surface antigen 1 (SAG1) was used as a marker of the surface.

To characterize the reactivity of anti-recombinant proteins mouse sera with the ESA and *T. gondii* tachyzoites, antisera against rTgRuvBL1, rTgRNase H1, and rTgRPS2 were used as the primary antibody for indirect ELISA and IFA. The ELISA results showed that TgRuvBL1, TgRNase H1, and TgRPS2 antisera were recognized by *T. gondii* tachyzoites-derived natural ESA (Figure 5C), indicating that these proteins were present in *T. gondii* ESA. Moreover, IFA using anti-TgRuvBL1, anti-TgRNase H1, or anti-TgRPS2 antibodies revealed that the three proteins were localized in different subcellular compartments in *T. gondii* tachyzoites, including the nucleus (TgRuvBL1) and the apical end (TgRNase H1 and TgRPS2; Figure 6).

### Detection of Three CAgs in the Sera of Infection Murine Models

**FIGURE 7:**
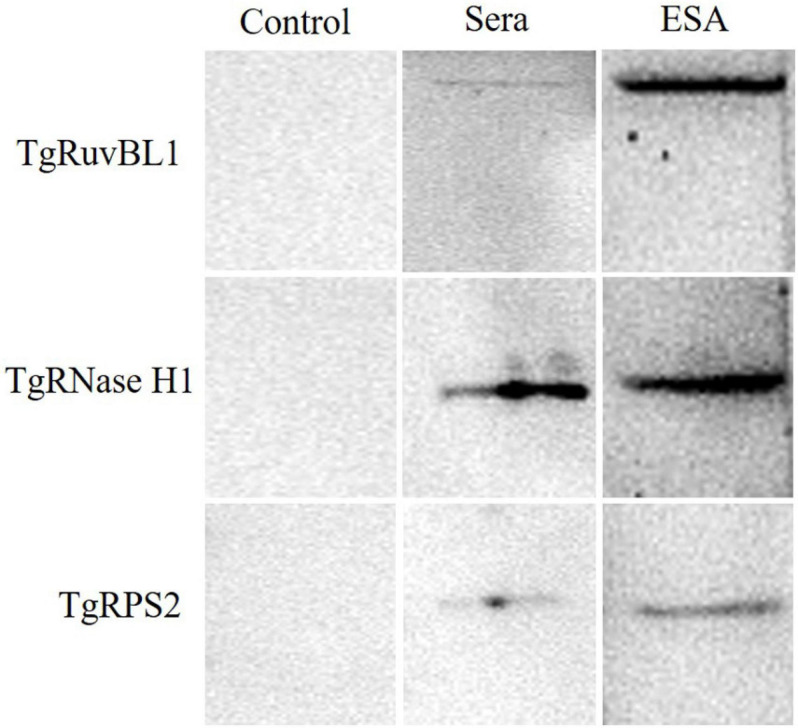
Western blotting of the recombinant proteins. Pooled serum samples from the control group were used as negative control. Pooled serum samples from the test group at 60 h were analyzed by western blotting at a dilution of 1:10. ESA was used as positive control.

Among the newly identified CAgs, the presence of TgRuvBL1, TgRNase H1, and TgRPS2 was verified in mouse sera. Pooled sera from the test and control groups were analyzed by immunoblotting, with ESA being used as positive control. The three CAgs were detected in the sera of mice with acute *T. gondii* infections (Figure 7).

### Protective Ability of Recombinant Proteins in Kunming Mice

**FIGURE 8:**
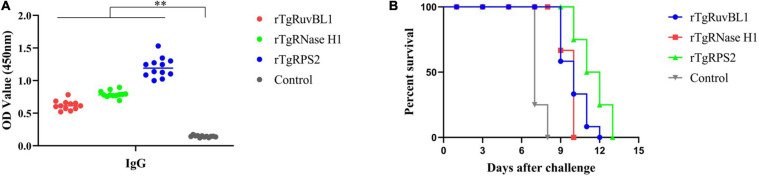
Survival curve of mice after challenge infection with *T. gondii* RH strain. **(A)** Levels of IgG in the sera of mice at week 2 after the final booster immunization. **(B)** Mice were challenged with 10^3^ tachyozoites of the RH strain injected intraperitoneally at week 2 after the third immunization. *p < 0.05 and **p < 0.01, compared with the control groups.

Significantly more IgG antibodies were detected in the sera of mice immunized with rTgRuvBL1, rTgRNase H1, or rTgRPS2 compared with control mice (Figure 8A). Figure 8B shows survival curves for the four groups of mice. Mice immunized with rTgRuvBL1, rTgRNase H1, or rTgRPS2 survived longer than control mice, all of which died within 8 days of challenge (10.0 ± 0.30, 9.67 ± 0.14, and 11.50 ± 0.34 vs. 7.25 ± 1.31 days; *P* < 0.05).

## Discussion

Detection of *T. gondii*-specific IgM and IgG antibodies is the main serological diagnostic method for acute toxoplasmosis. However, detection of IgG antibodies is not reliable for the diagnosis of acute infections, as they induce the production of low levels of IgG antibodies in acute infection and IgG positivity may be indicative of a previous infection. Moreover, IgM antibodies remain detectable for over a year. Therefore, even the presence of IgM antibodies does not necessarily indicate an acute infection (Elsheikha et al., 2020; Mcleod et al., 2020). ESA are the majority of the CAgs in sera from hosts with acute toxoplasmosis and they were proven to be a serologic marker for the diagnosis of acute toxoplasmosis and the infection route not affecting the detection of CAgs (Hafid et al., 1995; Abdollahi et al., 2009; Darcy et al., 2010; Xue et al., 2016). Particularly, they were shown to be diagnostic markers for cerebral toxoplasmosis in immunocompromised individuals (Meira et al., 2008; Meira et al., 2011). Thus, the identification of CAg components and screening of candidate diagnostic molecules can help improve the specificity and sensitivity of acute infection detection. However, only few studies have focused on the identification of CAgs.

In the current study, a murine model of acute *T. gondii* infection was established, which further demonstrated that CAgs could serve as markers of acute infection in mice in agreement with previous studies (Asai et al., 1987; Hassl et al., 1988; Chen et al., 2008; Azami et al., 2011; Wang et al., 2012; Xue et al., 2016). Moreover, CAgs were detected 3 h post-inoculation, which was consistent with the results of nested PCR. The levels of CAgs decreased during the following 3 to 24 h, and increased in the subsequent 24 to 60 h. The initial reduction might have been due to the host immune response and *T. gondii* in the bloodstream invading various organs.

Herein, 31 CAgs were identified in this study, among which, the diagnostic applications of SAG1, and ubiquitin-transferase domain-containing protein have been investigated (Felgner et al., 2015; Jiang et al., 2015). Moreover, some of the proteins identified, such as PLP1 (Kafsack et al., 2009), M2AP (Huynh et al., 2003), PI1 (Morris et al., 2002), Toxofilin (Poupel et al., 2000), Carbamoylphosphate synthetase (Fox et al., 2009), Myosin H (Graindorge et al., 2016), and CDPK9 (Long et al., 2016), have been shown to impair parasite progression through the lytic cycle, resulting in a loss of or weaker virulence when knocked-down. In addition, although most of the proteins have low reliability, some of them play important roles, such as ROP13 (Turetzky et al., 2010; Kang et al., 2019). Therefore, it is reasonable to believe that the CAgs identified in this study may contribute to the discovery of new virulence factors.

Furthermore, three CAgs – TgRuvBL1, TgRNaseH1, and TgRPS2 – were selected for prokaryotic expression to prepare murine polyclonal antibodies. Polyclonal anti-rTgRuvBL1, anti-rTgRNase H1, and anti-rTgRPS2 antibodies were recognized by natural ESA from *T. gondii* tachyzoites. Moreover, IFA data showed that they localize to two subcellular compartments, including the nucleus (TgRuvBL1) and the apical end (TgRNase H1 and TgRPS2). Of note, as both TgRNase H1 and TgRPS2 localize to the apical end, they may be involved in the invasion or egress of *T. gondii* tachyzoites. However, further research is warranted to explore this hypothesis. Moreover, these three proteins were found in the sera from mice with acute infection, which suggests that they may hold diagnostic potential for acute *T. gondii* infection and that their antibodies may be used in serological detection of CAgs. However, a large number of clinical trials are needed to verify whether they can be used in the diagnosis of acute infections. The present results also provide a theoretical basis for the continued development of rapid, highly specific, and accurate immunological diagnostic strategies.

Most CAgs are ESA that play important roles in inducing appropriate humoral and cellular immune responses against *T. gondii* (Hafid et al., 2005; Jongert et al., 2009; Costa-Silva et al., 2012; Chen et al., 2013; Daryani et al., 2014). Moreover, some proteins identified herein such as PLP1 (Yan et al., 2011), M2AP (Dautu et al., 2007), SAG1 (Mohamed et al., 2003), protease inhibitor PI1 (Cuppari et al., 2008), Histone H2A (Yu et al., 2020), Elongation factor 1-alpha (EF-1α; Wang et al., 2017), CDPK9 (Wang H. L. et al., 2016; Wang J. L. et al., 2016), HSP70 (Mohamed et al., 2003), protein disulfide-isomerase (Wang et al., 2013), and toxofilin (Song et al., 2017) elicit powerful specific immune responses, providing partial protection against acute or chronic *T. gondii* infection. Thus, we evaluated the protective ability of TgRuvBL1, TgRNase H1, and TgRPS2.

We showed that survival was prolonged in mice immunized with rTgRuvBL1, rTgRNase H1, or rTgRPS2, and then lethally challenged with *T. gondii* RH. However, all mice in our study died after challenge with *T. gondii* RH. The protective efficacy of these three recombinant proteins was weaker than that of other proteins, such as rTgEF-1 (14.53 ± 1.72 days; Wang et al., 2017), suggesting that they only weakly protected against infection by a highly virulent *T. gondii* strain. Although the demonstrated protection is not good enough for a vaccine, perhaps other proteins among the newly identified CAgs have better protective efficacy, similar to some other proteins identified in this study [e.g., PLP1 (Yan et al., 2011), EF-1α (Wang et al., 2017), among others].

## Conclusion

In summary, we identified 31 CAgs in the sera of mice infected with *T. gondii*. Among these CAg molecules, three novel proteins (TgRuvBL1, TgRNase H1, and TgRPS2) were successfully confirmed to be present in the sera of the infection mouse models. Although these proteins only showed a weak protection potential against a highly virulent *T. gondii* strain, they may represent potential diagnostic candidates for acute *T. gondii* infection.

## Data Availability Statement

The datasets presented in this study can be found in online repositories. The names of the repository/repositories and accession number(s) can be found below: ProteomeXchange Consortium via the PRIDE partner repository with the dataset identifier PXD021938 (https://www.ebi.ac.uk/pride/archive/projects/PXD021938).

## Ethics Statement

The animal study was reviewed and approved by The Animal Care and Use Committee of the Shanghai Veterinary Research Institute approved the study and the protocols complied with approved guidelines.

## Author Contributions

QL and QW participated in the experiment design and wrote the manuscript. QL and WJ analyzed the data. QL, QW, YC, MZ, and XG carried out the experiments. QL carried out bioinformatics analysis. All authors read and approved the final version of the manuscript.

## Conflict of Interest

The authors declare that the research was conducted in the absence of any commercial or financial relationships that could be construed as a potential conflict of interest.

## Supplementary Material

Supplementary Figure 1 SDS-PAGE analysis of CAg enriched and purified by immunoprecipitation. Circulating antigens enriched and purified by immunoprecipitation were analysed by SDS-PAGE (10 %). Lanes 1, 2, 3, and 4: immunoprecipitation supernatant. Due to the cracks in Lane 1 and Lane 2 is intercepted in the manuscript.

Supplementary File 1 List of CAgs proteins identified by LC-MS/MS after IP enrichment and purification with ESA antibodies.
