# Supplementary material for: Study on Circulating Antigens in Serum of Mice With Experimental Acute Toxoplasmosis
Source: Front Microbiol. 2021 Jan 18;11:612252. doi: 10.3389/fmicb.2020.612252 (PMC7848078; doi:10.3389/fmicb.2020.612252)
Supplement: Supplementary Figure 1 — SDS-PAGE analysis of CAg enriched and purified by immunoprecipitation. Circulating antigens enriched and purified by immunoprecipitation were analysed by SDS-PAGE (10 %). Lanes 1, 2, 3, and 4: immunoprecipitation supernatant. Due to the cracks in Lane 1 and Lane 2 is intercepted in the manuscript. [file Data_Sheet_1.docx]

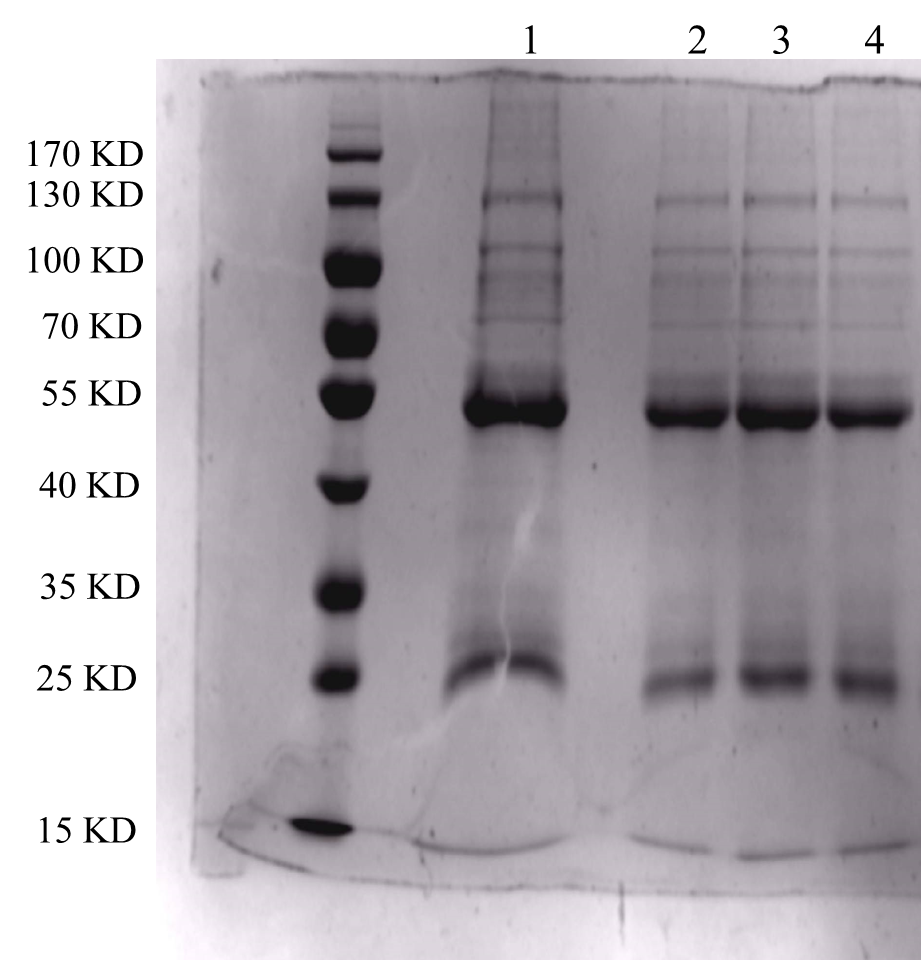


Fig. S1. SDS-PAGE analysis of CAg enriched and purified by immunoprecipitation. Circulating antigens enriched and purified by immunoprecipitation were analysed by SDS-PAGE (10 %). Lane 1,2,3,4: immunoprecipitation supernatant.

Due to the cracks in Lane 1, Lane 2 is intercepted in the manuscript.
